# Toward safer solid-state lithium metal batteries: a review

**DOI:** 10.1039/d0na00174k

**Published:** 2020-04-13

**Authors:** Zhenkang Wang, Jie Liu, Mengfan Wang, Xiaowei Shen, Tao Qian, Chenglin Yan

**Affiliations:** College of Energy, Key Laboratory of Advanced Carbon Materials and Wearable Energy Technologies of Jiangsu Province, Soochow University Suzhou 215006 China tqian@suda.edu.cn c.yan@suda.edu.cn

## Abstract

The solid-state lithium metal battery (SSLMB) is one of the most optimal solutions to pursue next-generation energy storage devices with superior energy density, in which solid-state electrolytes (SSEs) are expected to completely solve the safety problems caused by direct use of a lithium metal anode. Most previous work has mainly focused on improving the electrochemical performance of SSLMBs, but the safety issues have been largely ignored due to the influence of the stereotype that batteries with SSEs are always safe. In the actual research process, however, some potential dangers of SSLMBs have been gradually revealed, so extra attention should be paid to this issue. This minireview summarizes several aspects that could raise safety concerns and provides a brief overview of the corresponding solutions to each aspect. Finally, general conclusions and perspectives on the research of SSLMBs with ultra-high safety are presented.

## Abstract 2

This minireview summarizes several potential safety issues for solid-state lithium batteries, and the general conclusion and perspective on the research of solid-state lithium batteries with ultra-high safety are presented.

## Graphical abstract

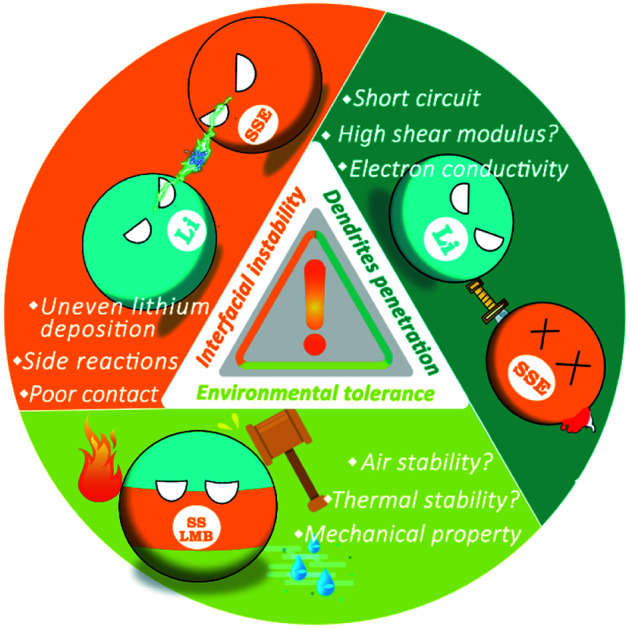

## 1. Introduction

At present, lithium ion batteries (LIBs) dominate energy storage devices and they have greatly innovated our lifestyle since their commercialization. However, the energy density of traditional LIBs has gradually reached a bottleneck.^1^ It is of great significance to develop energy storage systems with high energy density and safety to meet the sustainable development needs for energy and safe production.^2^ Using lithium metal as the anode material is strongly considered to be one of the most promising ways to improve energy density owing to its ultra-high theoretical capacity (3860 mA h g^−1^, ten times higher than graphite anodes in LIBs) and the lowest negative electrochemical potential (−3.04 V *vs.* SHE). However, the safety problem of lithium metal batteries is extremely intractable, which mainly arises from two aspects. First, lithium dendrites caused by uneven lithium ion deposition during the cycling process can cause an internal short circuit in the batteries.^3^ Second, existing conventional ethylene carbonate-based electrolytes are highly flammable and toxic, which pose a great safety risk to the lithium metal battery in practice. To realize the commercial application of lithium metal batteries in the future, the safety issues mentioned above must be solved first.

To overcome the flammable issue of the electrolyte, several strategies have been proposed, for instance, replacing the organic electrolytes with aqueous electrolytes,^4^ using special separators^5^ for an autonomic self-shutdown, or using electrolyte flame retardant additives.^6^ Meanwhile, scientists have also begun to pay attention to lithium metal anode protection, and considerable efforts have been devoted to solving the problems caused by lithium dendrites in the last few decades.^7^ Most studies mainly focused on constructing a strong artificial solid electrolyte interface or regulating the Li^+^ deposition by different methods, such as controlling the electric field area and building a lithophilic skeleton. Besides, the use of flammable liquid electrolytes is curtailed and SSEs are developed to improve battery safety. The high strength of SSEs could play a significant role in suppressing dendrites. Thus, SSEs may be the ultimate way to achieve high energy density and safety for batteries simultaneously.^8–11^

SSEs can be divided into three categories: inorganic SSEs, solid polymer electrolytes (SPEs), and their hybrids, Table 1 summarizes most existing SSEs and their properties such as lithium ion conductivity and their physical and chemical properties. Inorganic SSEs mainly include oxides (such as Li_7_La_3_Zr_2_O_12_ (LLZO) and Li_3.3_La_0.56_TiO_3_), sulfides (such as Li_2_S–P_2_S_5_ and Li_10_GeP_2_S_12_), hydrides (such as LiBH_4_), and other types. In all inorganic types of SSEs, the oxide and sulfide types currently have the most practical potential due to their high ionic conductivity (10^−4^ to 10^−2^ S cm^−1^),^12^ which has reached or even exceeded that of standard non-aqueous electrolytes, such as Li_6.75_La_3_Zr_1.75_Te_0.25_O_12_ (8.7 × 10^−4^ S cm^−1^)^13^ and Li_10_GeP_2_S_12_ (LGPS, 1.2 × 10^−2^ S cm^−1^).^14^ SPEs consist of a polymer with a polar group (–O–, =O, –N–, *etc.*), such as polyethylene oxide (PEO), polymethyl methacrylate (PMMA), or poly(vinylidene fluoride) (PVDF). Among them, PEO is currently the most widely used.

**Table 1:** Summary of most existing SSEs and their properties

Type	Conductivity (S cm^−1^)	Physical properties	Chemical properties
**Inorganic SSEs**			
*Oxides*			
Li_7_La_3_Zr_2_O_12_^82^	10^−5^ to 10^−3^	• High mechanical strength	• Thermal stability
Li_3.3_La_0.56_TiO_3_^83^		• Non-flexible	• Air stability
Li_6.4_La_3_Zr_1.4_Ta_0.6_O^13^		• High conductivity	• High electrochemical stability
Li_1.5_Al_0.5_Ge_1.5_(PO_4_)_3_,^71^*etc.*			
*Sulfides*			
Li_2_S–P_2_S_5_^57^	10^−4^ to 10^−2^	• Good mechanical strength	• Sensitive to moisture
Li_10_GePS_12_^14^		• High conductivity	• Low oxidation stability
Li_10_SnP_2_S_12_,^84^*etc.*			• Poor compatibility with lithium metal
*Hydrides*			
LiBH_4_^88^	10^−8^ to 10^−5^	• Good mechanical strength	• Sensitive to moisture
LiBH_4_–LiX (X = Cl, Br, or I)^88^*etc.*		• Relatively low conductivity	• Stable with lithium metal
			• Low oxidation stability

**SPEs**			
PEO^85^	10^−7^ to 10^−3^	• High mechanical flexibility	• Limited thermal stability
PMMA^86^		• Low mechanical strength	• Stable with lithium metal
PAN^87^		• High ductility	• High oxidation stability
PVDF^48^			• Air stability
PVC^86^*etc.*			

**Fig. 1:**
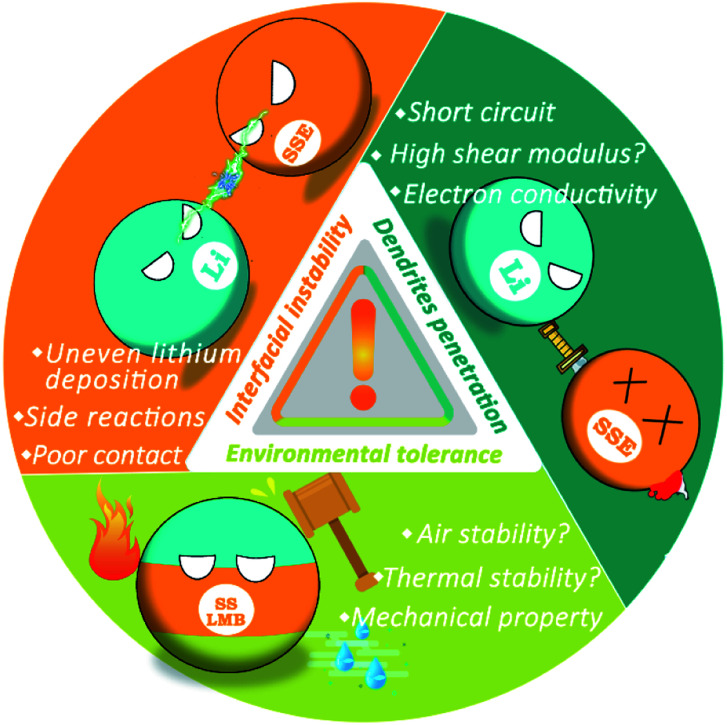
Three potentially dangerous aspects of SSLMBs.

However, LMBs with SSEs still face serious safety risks since the internal reaction and ion transmission mechanisms become more complex, which mainly arises from three aspects (Fig. 1): (1) in addition to high mechanical strength, dendrite growth is related to many factors. SSEs in SSLMBs cannot effectively suppress the penetration of lithium dendrites, which not only reduce the performance of the battery but also lead to severe safety concerns. (2) The instability of the interface between SSEs and the electrode cause continuous chemical reactions, which in turn increase the instability of the entire battery system. (3) It is difficult to achieve absolute safety for SSLMBs in the face of external environmental influences such as mechanical forces or air instability. The purpose of this minireview is to analyze the causes of the safety issues of SSLMBs in detail based on the three aspects mentioned above and to describe recently proposed strategies to render SSLMBs safe. The safety issues of SSLMBs are currently being paid a relatively low degree of attention, we believe that this minireview will contribute to the future development of a SSLMB with excellent safety and high energy density.

## 2. Dendrites penetration in SSEs

### 2.1 Dendrites penetration in inorganic SSEs

According to the theory proposed by Monroe and Newman, lithium dendrites can be effectively suppressed when an SSE exhibits a high shear modulus of approximately twice that of lithium (about 4.2 GPa).^15,16^ In the present case, the shear modulus of sulfide type and oxide garnet type SSEs has reached this criterion so that SSEs are considered to be a promising way to resolve the short circuit problem ultimately and to greatly improve the safety of lithium batteries.

**Fig. 2:**
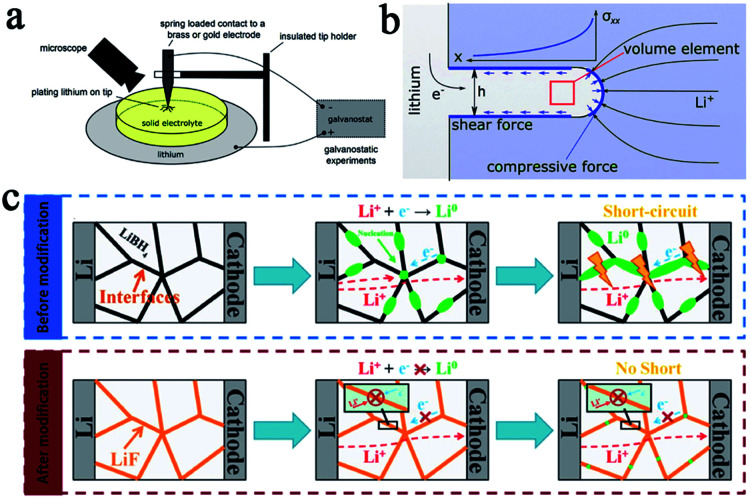
(a) A schematic of an apparatus for Li plating on a metal electrode in contact with a solid electrolyte and (b) a simplified schematic of a Li filament in a solid electrolyte matrix. This model predicts a maximal stress at the filament tip, adapted with permission from ref. 17, Wiley-VCH Verlag GmbH & Co., Copyright 2017. (c) Li dendrite formation in a solid electrolyte before modification and Li dendrite suppression mechanism in a modified electrolyte system, adapted with permission from ref. 19, Wiley-VCH Verlag GmbH & Co., Copyright 2019.

However, multiple recently reported research reports showed that solid state batteries with inorganic SSEs and lithium metal anodes still experience a short circuit. Some research groups have studied the mechanism of lithium dendrite penetration into inorganic SSEs. Chiang's group used *in situ* and *ex situ* optical microscopy (Fig. 2a) to investigate the diffusion mechanism of lithium dendrites in four types of SSEs, including glassy LPS, β-Li_3_PS_4_ and polycrystalline and single-crystal Li_6.4_La_3_Zr_1.4_Ta_0.6_O_12_ (LLZTO), and developed an electrochemomechanical model to describe the penetration behavior of lithium dendrites in SSEs (Fig. 2b).^17^ They came to the conclusion that the shear-modulus was not the determining factor to suppress dendrites for those inorganic SSEs. The lithium infiltration can be effectively reduced by reducing the defect size and improving the density of SSEs. Wang's group conducted a deeper research of the dendrites' formation mechanism in inorganic SSEs. They studied in detail the formation of dendrites in different inorganic SSEs (LLZO, LPS, and LiPON) by using operando neutron depth profiling and suggested that the electronic conductivity of SSEs was another critical factor of the growth of lithium dendrites.^18^ Due to the high electronic conductivity of LLZO and LPS, lithium ions can directly capture electrons inside the SSEs during charging and are then deposited as lithium dendrites, causing short circuits and greatly increasing the risk of batteries. This statement is more convincing now and some other research reports have further confirmed it. For example, Sun's group also has demonstrated that the formation of lithium dendrites in LiBH_4_ was mainly due to its relatively high conductivity through experiments and theoretical calculations.^19^ Based on this principle, LiF with a gap filling ability and low electron conductivity was introduced into LiBH_4_, which significantly enhanced the stability and cycle life of the batteries. The mechanism of inhibiting lithium penetration by LiF in SSE is shown in Fig. 2c. The modified SSE was assembled into a lithium metal battery using TiS_2_ as the cathode, and the battery exhibited a reversible capacity of 137 mA h g^−1^ after 60 cycles at 0.4C.

### 2.2 Dendrites penetration in SPEs

For LMBs with SPEs, the dendrite problem is still a formidable challenge. On the one hand, in general, SPEs have a lower shear modulus. For example, the shear modulus of PEO is only about 0.1 MPa, so that lithium dendrites can easily pierce the electrolyte layer and cause short circuits. On the other hand, existing research shows that the formation of lithium dendrites is caused by Li^+^ concentration gradients in SPEs. Conventional SPEs, such as PEO-based SPEs, can conduct both Li^+^ and its counter anions (TFSI^−^ and PF_6_^−^). During discharging, the anion and cation move in opposite directions in the polymer matrix and the ion transference number (*t*) satisfies eqn (1), shown below;^26^ however, the anions tend to accumulate at the anode side and block the surface of the electrode, which leads to severe polarization and heterogeneous lithium deposition.^27,28^

1 *t*_Li^+^_ + *t*_anion_ = 1

**Fig. 3:**
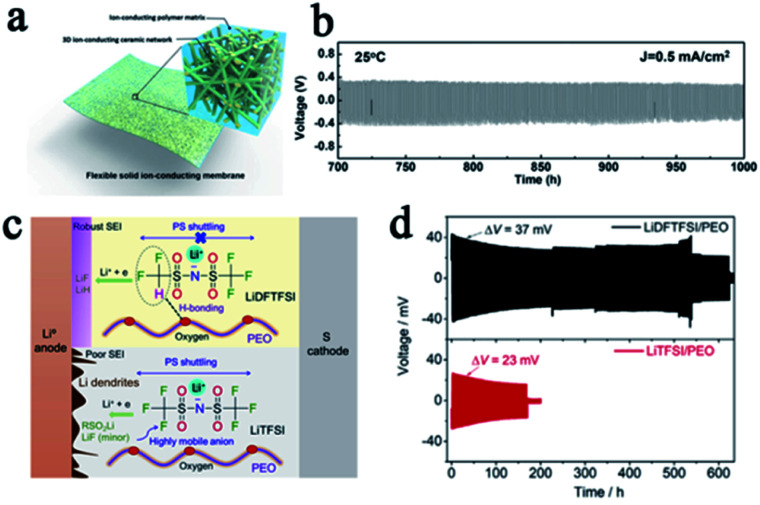
(a) A schematic of the hybrid solid-state composite electrolyte and (b) the voltage profile of the continued lithium plating/stripping cycling with a current density of 0.5 mA cm^−2^ at 25 °C, adapted with permission from ref. 23, *Proc. Natl. Acad. Sci. U. S. A.*, American Institute of Physics, Copyright 2016. (c) The role of LiDFTFSI and LiTFSI in PEO and (d) the performance comparison of lithium symmetrical batteries with different SPEs, adapted with permission from ref. 32, Elsevier, Copyright 2019.

It is accepted that composite electrolytes, including inorganic–polymer composite electrolytes and polymer–polymer composite electrolytes, can be a promising way to tackle the problem of the low shear modulus of SPEs.^20–22^ In inorganic–polymer composite electrolytes, commonly used inorganic fillers are classified into inert fillers such as SiO_2_, TiO_2_, and Al_2_O_3_, and active fillers which are mainly Li^+^ conductors such as LLZO and LGPS. Both kinds of inorganic fillers can improve the mechanical strength of SPEs and impose a certain inhibitory effect on lithium dendrites. For example, Fu *et al.* prepared LLZO nanofibers and then made it into a composite with PEO to obtain an SSE with three-dimensional ion transmission channels and a high shear modulus. The batteries with this SPE were not short-circuited even after more than 1000 hours of cycling (Fig. 3a and b).^23^ Recently, Cui's group proposed a design strategy for ultra-thin, high-performance polymer–polymer composite SPEs for LMBs. They filled PEO/LiTFSI into the polyimide film, which greatly enhanced the shear modulus (0.1 to 850 MPa) and prevented the penetration of lithium dendrites.^24^ Molecular structure design is another way to improve the properties of SPEs. Zeng's group designed a polyether-acrylic interpenetrating SPE which combines the flexibility of polyether with the rigidity of polyacrylic, exhibiting a high ionic conductivity (0.22 mS cm^−1^) and a high shear modulus (about 12.0 GPa). The batteries with this SPE exhibited excellent stability and safety performance, with no formation of lithium dendrites after 200 cycles and no circuit appears even after the battery pouch was cut.^25^

In addition to increasing the mechanical strength of SPEs to inhibit lithium penetration, eliminating the growth of lithium dendrites in SPEs from the source is even more important. According to Newman and Monroe's simulations,^29,30^ if the *t*_Li^+^_ is close to unity, there will be no Li^+^ concentration gradient in the electrolyte, thus promoting the uniform lithium deposition even at a relatively large current density. However, both Li^+^ and their counter anions are mobile in conventional SPE systems, and the *t*_Li^+^_ is usually less than 0.5 since the motion of Li^+^ is highly coupled with the Lewis basic sites in the polymeric matrix. Single Li^+^ conducting SPEs are one of the most promising ways to solve this fundamental problem.^26^ In single Li^+^ conducting SPEs, the anions were covalently attached to the polymeric matrix so that the *t*_anion_ was greatly suppressed and the *t*_Li^+^_ was greatly enhanced up to unity, which reduced the concentration gradient in SPEs and alleviated the driving force for dendrite formation.^29,31^ This concept has also been proven to be a potential way to suppress lithium dendrite formation by most previous reports. Single-ion SPEs include different types, such as blend polymers, random copolymers, block copolymers, *etc.* This also means that people can design the single-ion SPE in a variety of ways. Some recently reported single-ion SPEs are listed in Table 2, and their anionic center, ionic conductivity, and lithium ion transference number are summarized. All of these SPEs effectively inhibit the penetration of lithium dendrites and protect SSLMBs from short circuiting. Michel Armand also proposed a novel method to reduce the *t*_anion_ by designing the lithium salt anion composition. They replaced a fluorine atom in [N(SO_2_CF_3_)_2_]^−^ (TFSI^−^) with a hydrogen atom, and obtained [N(SO_2_CF_2_H)(SO_2_CF_3_)]^−^ (DFTFSI^−^). The CF_2_H moiety can form a hydrogen bond with the oxygen in PEO and the strong hydrogen bonding interaction is beneficial for restricting anionic mobility (Fig. 3c). This SPE effectively promoted uniform lithium deposition and greatly enhanced the stability of batteries (Fig. 3d).^32^

**Table 2:** Physicochemical properties of some recently reported single Li^+^ conducting SPEs with various anionic centers^a^

Type of electrolyte	Anionic center	*σ*_Li^+^_ (S cm^−1^)	*t*_Li^+^_	Ref.
Blend polymer	–SO_2_N^(−)^SO_2_CF_3_	1.1 × 10^−4^ (25 °C)	0.86 (25 °C)	33
	–SO_2_N^(−)^SO_2_CF_3_	7.33 × 10^−5^ (60 °C)	0.84 (60 °C)	35
	–SO_2_N^(−)^SO_2_-ph	3.08 × 10^−4^ (25 °C)	0.97 (80 °C)	36
	–SO_2_N^(−)^SO_2_F	1.43 × 10^−5^ (60 °C)	0.90 (60 °C)	37
	–SO_2_N^(−)^SO(=NSO_2_CF_3_)CF_3_	6.92 × 10^−5^ (70 °C)	0.91 (60 °C)	38
Random copolymer	–NH_2_(CN_4_)–	1 × 10^−4^ (80 °C)	0.94 (80 °C)	45
	–SO_2_N^(−)^SO_2_CF_3_	1.8 × 10^−4^ (30 °C)	0.91 (30 °C)	34
Block copolymer	–BO_3_^−^	4.95 × 10^−6^ (30 °C)	0.88 (30 °C)	41
	–BO_4_^−^	1.47 × 10^−3^ (25 °C)	0.89 (25 °C)	43
Triblock copolymer	–SO_3_^−^	3.0 × 10^−5^ (90 °C)		39
	–SO_3_^−^	1.45 × 10^−4^ (25 °C)	0.92 (25 °C)	40
	–BO_4_^−^	1.32 × 10^−3^ (25 °C)	0.92 (25 °C)	42
	–CO_2_^−^	1.61 × 10^−4^ (80 °C)	0.86 (80 °C)	44

a Blend polymer: a physical mixture of homopolymers or copolymers with different structures. Random copolymer: a polymer comprising different repeating units with random distribution. Block copolymer: a polymer containing alternating segments of different polymer compositions, linked together through their reactive ends. Triblock copolymer: a polymer composed of three blocks of homopolymers in a linear sequence.

Theoretically, it is well known that a perfect SSE without any defects can suppress the formation of lithium dendrites. However, both inorganic SSEs and SPEs are still facing the threat of lithium dendrite penetration, which has been widely observed in different electrolytes, consequently resulting in a battery short-circuit. More and more researchers began to pay attention to this problem. In fact, we can also take inspiration from the methods of inhibiting dendrites in liquid batteries and apply it to solid-state battery systems, for example, by introducing electrochemically and mechanically stable *ex situ* coatings as the artificial SEI layer to separate the SEI layer from dendrite growth toward a uniform deposition,^94^ by confining lithium in a conductive lithiophilic framework,^95^ by controlling the current density and areal capacity loadings to realize a gentle lithium deposition,^7^*etc.* Up to now, the mechanism of dendrite generation in SSEs has not been fully understood, but it is certain that any progress in the study of this fundamental problem will further significantly improve the safety of SSLMBs.

## 3. Interfacial stability problems

In SSLMBs, it is difficult for the interface between the lithium anode and SSE to maintain long-term stability since the SSE is prone to side reactions when in contact with the lithium metal anode which has ultra-low electrochemical potential and ultra-high chemical reactivity.^46^ The side reaction and the formation of reactants between the lithium anode and SSEs will finally result in the following serious consequences: (1) continuing to consume the lithium metal and SSE, and accelerating battery failure; (2) causing volume changes inside the battery and threatening the structural strength of the electrolyte; (3) increasing the disorder of the electric field and providing ample space for the initial growth of lithium dendrites, which could create localized stress and result in fracture. For a perfect interface, there should first be full contact between the SSE and electrode, and then the metastable layer formed at the interface should be ionic but not electron conductive,^46^ so as to stop further side reactions and prevent Li^+^ from capturing electrons directly in SSE to form dendrites.

**Fig. 4:**
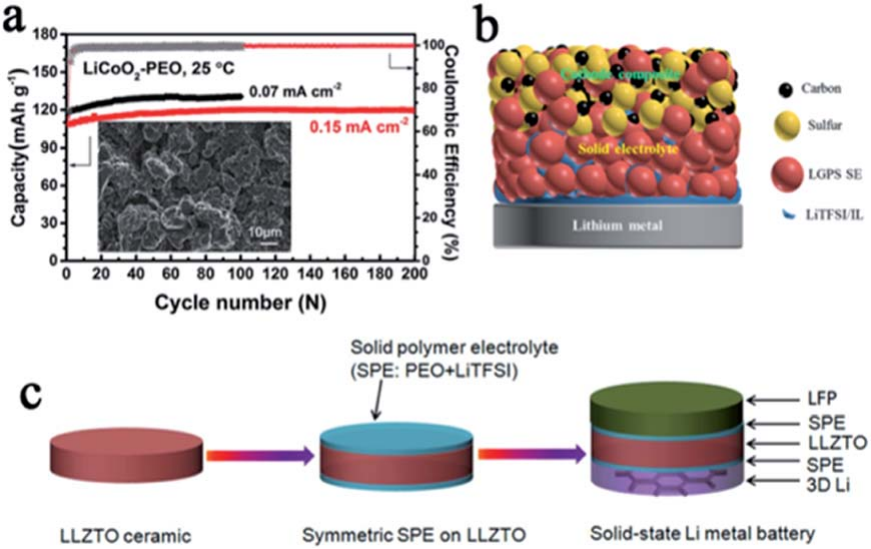
(a) Cycling performance of a LiCoO_2_–PEO‖Li battery with PVDF–LiFSI as the SPE, adapted with permission from ref. 48, Wiley-VCH Verlag GmbH & Co., Copyright 2019. (b) A schematic diagram of the interface regulation of an ionic liquid, adapted with permission from ref. 51, American Chemical Society, Copyright 2019. (c) A schematic illustration of the fabrication process of the SPE interface layer with a 3D Li anode for SSLMBs, adapted with permission from ref. 54, Elsevier, Copyright 2018.

It is undoubtedly a good strategy to design and modify the interface layer between the SSE and electrode.^47^ Through systematic experiments combined with DFT calculations, Nan *et al.* found that the *in situ* formed nanoscale interface layer between the PVDF-based SSE and lithium anode was highly stable and could effectively suppress dendrite growth.^48^ This interface layer enables high performance of both the Li‖Li symmetric battery (stable over 2000 h cycling, 0.1 mA cm^−2^) and the LiCoO_2_‖Li battery (almost no capacity decay after 200 cycles, 0.15 mA cm^−2^) (Fig. 4a). Thanks to many superior safety properties such as excellent thermal stability, high Li^+^ conductivity, and excellent wettability, ionic liquids are considered to act as trace surface modifiers between SSEs and electrodes to solve interface problems, thereby improving battery safety.^49,50^ For example, Yang *et al.* promoted LiTFSI/PYR_13_TFSI as a trace wetting agent, which improved the contact and stability between lithium and LGPS greatly (Fig. 4b), and thus successfully enhanced the cycle stability of the battery.^51^ Stable lithium stripping/plating performance over 1000 h at 0.1 mA cm^−2^ was obtained, and the interfacial impendence was reduced to 142 Ω cm^−2^. Constructing an intermediate buffer layer is also a very effective approach to reduce the interfacial resistance and stabilize the lithium anode, and this layer should be stable to lithium and conductive to Li^+^.^46,52^ Yao *et al.* designed a double-layer LGPS–70Li_2_S–29P_2_S_5_–1P_2_O_5_ SSE, with 70Li_2_S–29P_2_S_5_–1P_2_O_5_ as the buffer layer in contact with the lithium anode, showing good contact and stability.^53^ Chi's group introduced a PEO-based SPE interface layer in LLZTO to better settle the interface contact and stability issues, while using a 3D lithium anode, which could reduce the local current density and increase the number of Li^+^ deposition sites, to suppress dendrite growth (Fig. 4c).^54^ Thanks to this ingenious design, the symmetric battery exhibits an excellent cycle stability after 700 h.

In addition to the corresponding design and regulation of the SSE interface, it is also an excellent strategy to design and regulate the lithium metal anode, such as by using a lithium alloy anode to replace the pure lithium anode. Lithium–indium alloys have long been found to stabilize the interfacial layer and inhibit the formation of lithium dendrites when matching with sulfide type SSEs.^55,56^ Philipp Adelhelm *et al.* studied the phase formation, redox potentials, and interface stability of a Li–In alloy anode in a solid state battery with β-Li_3_PS_4_.^57^ The results show that different Li–In ratios will form different alloy phases, and when the ratio was 1 : 1.26, the overpotential of Li^+^ stripping/plating was as low as 12 mV over 200 h and no significant change was observed. Some other lithium alloys have also been shown to stabilize and wet the interface between the SSE and anode, such as a Li–Mg alloy,^58^ Li–ZnO alloy,^59^ Li–Al alloy,^60^ Li–C alloy,^61^*etc.*

In addition, the incompatibility between the SSE and electrode (both anode and cathode) is another important reason that leads to interfacial instability, especially in some rigid SSEs such as ceramic SSEs. The poor solid–solid contact greatly increases the internal impendence of the battery, which not only deteriorates the structure of the interface, but also causes the battery to overheat during rapid charging. Except for the methods mentioned above (building flexible intercalation or using interface wetting agents) used to achieve good interface contact, some other approaches are also useful, such as using a lithiophilic intercalation layer,^90^ reducing the surface tension of molten lithium,^61^ or constructing an electrode with a 3D structure.^92,93^ In particular, *in situ* polymerization should be given more attention. Lynden A. Archer used aluminium triflate as an initiator to initiate open-loop polymerization of DOL. The *in situ* formed SPE had excellent mechanical and chemical stability, maintained good interface contact with the battery electrode, and finally achieved high room temperature ionic conductivity and low interface impedance.^89^ This technology is convenient, effective, and exhibits great potential to solve poor contact of both anode and cathode to SSE.

In summary, the cause of the interface problems between the SSE and active lithium anode is very complicated, and it poses a great threat to the safety and stability of SSLMBs. Due to the unique physicochemical properties of different SSEs, strategies based on composite, multilayer or asymmetric SSEs, which combine the advantages of different SSEs, are investigated to solve the interface challenge. Besides, the development of *in situ* electrochemical methods based on the interface between the SSE and electrode can be used to further elucidate the mechanism of the interface failure, which is of great significance for achieving a safer SSLMB.

## 4. Environmental tolerance

**Fig. 5:**
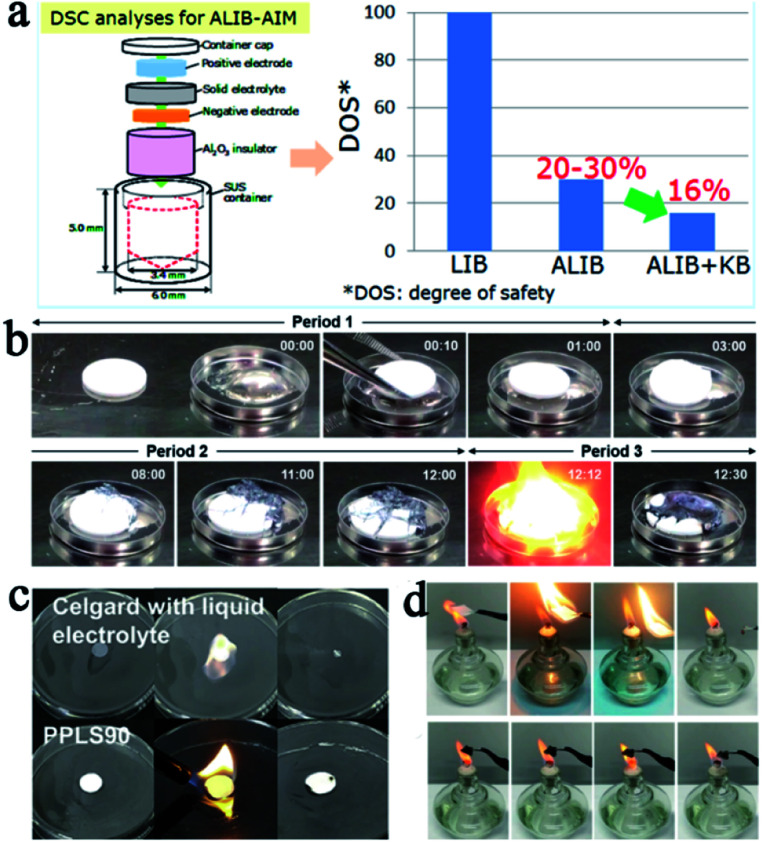
(a) Schematic of the procedure of the all-inclusive microcell followed for DSC analyses and the test results of the battery with liquid and solid electrolytes, adapted with permission from ref. 62, American Chemical Society, Copyright 2017. (b) Sequential images as a function of time for contact of sintered LAGP pellet and melted Li metal at 200 °C in the glovebox, adapted with permission from ref. 71, American Chemical Society, Copyright 2017. (c) The flammability test of the Celgard separator and PPL90 membrane, adapted with permission from ref. 75, Elsevier, Copyright 2019. (d) The flammability test of the Celgard separator, PEO, and HVTPE, adapted with permission from ref. 76, American Chemical Society, Copyright 2019.

It is generally accepted that SSEs are safer compared with conventional liquid electrolytes owing to their better thermal and environmental stability. However, from a practical point of view, SSLMBs are not so satisfactory. Mukai *et al.* used a differential scanning calorimeter to study the heat generation behavior of all solid-state LIBs with LLZNO as the electrolyte. The conclusion was reached that the SSE reduced heat production of the liquid electrolyte to 30%, but it cannot be absolutely secure (Fig. 5a).^62^ For SSLMBs, the situation could be even worse due to the high reactivity of the lithium metal anode.

The ductility of inorganic SSEs is poor. They are easily broken or even shattered when subjected to uneven external forces (such as being squeezed, hit, *etc.*), which can easily cause a short circuit and serious safety problems. Besides, some inorganic SSEs are unstable in air, once the battery package is damaged by external force, subsequent reactions will increase the degree of danger.^81^ For example, sulfide type SSEs, a class of SSEs with great commercial potential thanks to their outstanding ionic conductivity, once combined with water molecules in the air, will not only cause fatal damage to the battery's performance, but also will release toxic hydrogen sulfide and present a potential risk. Therefore, improving the air stability and ductility of the electrolyte can improve the safety and is conductive to a better commercialization of SSLMBs. In terms of the air-stability of sulfide type SSEs, previous research^63^ has indicated that the air stability of Li_2_S–P_2_S_5_ can be improved by controlling the ratio of Li_2_S and P_2_S_5_, and the best stability could be obtained when the ratio was 3 : 1 due to the higher content of PS_4_^3−^, whose reactivity with water molecules was lower than S^2−^ and P_2_S_7_^4−^. In addition, some other oxides in the sulfide SSEs instead of sulfide conductors can also improve the stability of sulfide SSEs such as Li_2_O,^64^ P_2_O_5_,^65^*etc.* Hayashi dispersed the metal oxide (M_*x*_O_*y*_, M = Fe, Zn and Bi) in the Li_2_S–P_2_S_5_ by ball milling, which not only improved stability of the electrolyte, but also absorbed hydrogen sulfide through a spontaneous reaction (M_*x*_O_*y*_ + H_2_S = M_*x*_S_*y*_ + H_2_O), further increasing the safety of the batteries.^66^ Compounding inorganic SSEs with polymers is one of the most effective ways to improve their flexibility.^67–70^ For thermal stability, most inorganic SSEs are nonflammable and exhibit high thermal stability. However, some research results show that some inorganic SSEs cannot maintain good thermal stability once thermal runaway occurs. For example, Chung's group brought a piece of sintered Li_1.5_Al_0.5_Ge_1.5_(PO_4_)_3_ (LAGP) ceramic directly into contact with molten lithium (about 200 °C) in a glove box. The rapid dynamic reaction at high temperature drives the SSE structure to collapse and rapidly decompose in oxygen, inducing further rigorous thermal runaway (Fig. 5b).^71^

**Fig. 6:**
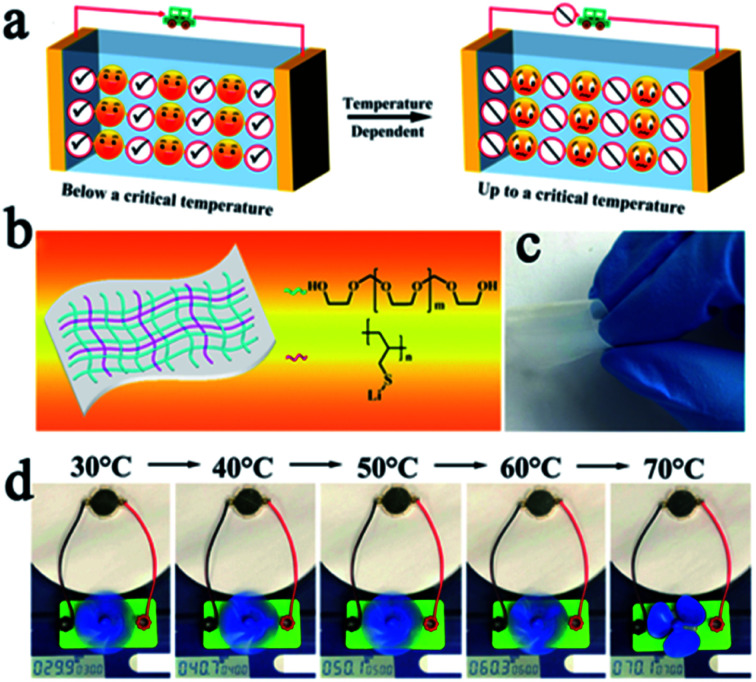
(a) The mechanism of thermal response polymer to prevent thermal runaway. (b) The schematic illustration of the composition of thermal-responsive SPE and (c) its optical image. (d) Photographs of a small electric fan powered by one SSLMB at different temperatures. Adapted with permission from ref. 80, American Chemical Society, Copyright 2019.

SPEs have strong ductility and flexibility, and existing mainstream PEO-based SPEs are more non-flammable than liquid electrolytes, but they still show the possibility of burning when the battery undergoes thermal runaway and cannot be absolutely safe. Compositing PEO or other flammable SPEs with fire retardant or non-combustible materials may be an effective strategy to solve this problem.^23,24,72–74^ Song *et al.* used PVDF-HFP/PEO as an organic matrix, LAGP and a solvate ionic liquid as the filler to develop a high-performance hybrid SSE (named PPLS90) with outstanding environmental and thermal stability.^75^ The PPLS90 can be exposed to a flame for 30 s and does not ignite, whereas the Celgard separator with a liquid electrolyte ignited easily (Fig. 5c). Yan's group reported high stability and safety SPEs by using ring-opening polymerization of fluoroethylene carbonate (FEC) as the polymer matrix and lithium difluoro(oxalato)borate (LiDFOB) as a lithium salt.^76^ This SPE not only can stay stable at an ultra-high voltage (4.9 V), but also does not burn after ignition (Fig. 5d). The methods mentioned above only work after the battery has undergone thermal runaway, if the thermal runaway of the battery can be suppressed at the source, this problem will be better solved. Thermally responsive polymers with unique thermal properties such as phase transformation, sol–gel transitions, and internal reactions, are expected to be an effective strategy for preventing thermal runaway and this concept has been proven to be able to effectively improve the safety of liquid LIBs.^77–79^ The mechanism of thermal response polymers to prevent thermal runaway is shown in Fig. 6a. Yan *et al.* reported a novel high-ionic-conductive thermo-responsive SPE which was constructed by the copolymerization of poly(1,3-dioxolane) and poly(lithium allyl-sulfide) (Fig. 6b and c).^80^ This SPE shows an ingenious, autonomic response to temperature change. When the operation temperature explodes to a certain threshold (70 °C), the thermoresponsive SPE will cut off ion transmission channels and end the thermal runaway by forcibly stopping the battery operation (Fig. 6d).

The development tendency of batteries should be catered for with an integrated, isolated and convenient method. Recently, some research based on air-stable lithium anodes has been reported, and the assembly of batteries in air is very compelling,^91^ as well as the assembly of SSLMBs. However, the decomposition of some SSEs after meeting water or oxygen still hinders the development of SSEs that are stable in the air. There remains much work to do to overcome this challenge. Furthermore, there is little research on all solid-state thermal-responsive polymer batteries, but it is undeniable that this is an area worthy of further research.

## 5. Summary and perspective

Incorporating a lithium metal anode into batteries is considered to be a promising way to greatly enhance the energy density, but the safety issues that come with lithium dendrites hinder their practical application. SSEs can avoid the safety problem and achieve high energy density for batteries, holding great potential for the application of lithium metal anodes. Until now, extensive progress has mainly focused on enhancing the ionic conductivity of bulk SSEs and improving performance of SSLMBs (reduced interface impedance, improved cycle stability, *etc.*) at room temperature. Actually, the safety design and requirements for battery products should be the highest priority in the entire battery system. Although SSLMBs have a brilliant future, the safety of SSLMBs has received relatively little attention. Although SSEs have greatly improved battery safety, they still cannot reach the ideal ultimate safety and there is still a long way to go to achieve this goal.

From a safety point of view, we have taken some classic SSEs as examples to give a brief overview of the potential unstable and dangerous factors of SSLMBs. Among the three potential issues mentioned above, the lithium dendrite problem remains the biggest threat to SSLMB safety issues. The traditional view that a high shear modulus of SSEs is needed to suppress lithium dendrites should be changed. Conquering lithium dendrites should start from multiple aspects, including interface engineering regulation, the modification of the electrolyte itself, and the lithium metal anode. It is not difficult to find out that almost no SSEs can achieve both high performance and high safety performance. They usually have an excellent performance in some aspects, but there are always unsatisfactory aspects in other areas regardless of whether they are inorganic SSEs or SPEs. Composite SSEs may be the ultimate path to achieve a safe SSLMB. The introduction of polymers can make up for the shortcomings of inorganic electrolytes such as instability when matched with lithium, higher electronic conductivity, and poor flexibility. Inorganic SSEs can make up for the defects of SPEs in terms of ionic conductivity and poor mechanical strength, and can reduce the possibility of burning of polymer electrolytes. Besides, little research has been done on all solid-state thermal-responsive SPEs yet, and this aspect should be paid enough attention since its fuse-like effect will bring the safety of batteries to a new level. Finally, discussion about the relevant testing standards for SSLMB safety should also be on the agenda. We believe that SSLMBs with both a high energy density and extremely high safety will eventually be realized through the cooperation of chemistry, energy, materials, engineering, battery management, and other fields.

## Conflicts of interest

There are no conflicts to declare.

## Supplementary Material
